# Latest Developments in Edible Coatings on Minimally Processed Fruits and Vegetables: A Review

**DOI:** 10.3390/foods10112821

**Published:** 2021-11-16

**Authors:** Amalia Carmen Miteluț, Elisabeta Elena Popa, Mihaela Cristina Drăghici, Paul Alexandru Popescu, Vlad Ioan Popa, Oana-Crina Bujor, Violeta Alexandra Ion, Mona Elena Popa

**Affiliations:** 1 Faculty of Biotechnology, University of Agronomic Sciences and Veterinary Medicine of Bucharest, 011464 Bucharest, Romania; amaliamitelut@yahoo.com (A.C.M.); mihaeladraghici38@gmail.com (M.C.D.); paul.popescu@biotehnologii.usamv.ro (P.A.P.); monapopa@agral.usamv.ro (M.E.P.); 2 Research Center for Studies of Food Quality and Agricultural Products, University of Agronomic Sciences and Veterinary Medicine of Bucharest, 011464 Bucharest, Romania; popa.ivlad@yahoo.com (V.I.P.); oana.bujor@qlab.usamv.ro (O.-C.B.); violeta.ion@qlab.usamv.ro (V.A.I.)

**Keywords:** edible coatings, fruits, vegetables, shelf life, functional coatings

## Abstract

The food industry nowadays is facing new challenges in terms of sustainability and health implications of packaging and processing techniques. Due to their desire for new and natural products coupled with changes in lifestyle, consumers are looking for food products that have been less processed but possess longer shelf life and maintain nutritional and sensorial proprieties during storage. These requirements represent real challenges when dealing with highly perishable food products, such as fruits and vegetables. Thus, in recent years, edible coatings have been intensively developed and studied because of their capacity to improve the quality, shelf life, safety, and functionality of the treated products. Edible coatings can be applied through different techniques, like dipping, spraying, or coating, in order to control moisture transfer, gas exchange, or oxidative processes. Furthermore, some functional ingredients can be incorporated into an edible matrix and applied on the surface of foods, thus enhancing safety or even nutritional and sensory attributes. In the case of coated fruits and vegetables, their quality parameters, such as color, firmness, microbial load, decay ratio, weight loss, sensorial attributes, and nutritional parameters, which are very specific to the type of products and their storage conditions, should be carefully monitored. This review attempts to summarize recent studies of different edible coatings (polysaccharides, proteins, lipids, and composites) as carriers of functional ingredients (antimicrobials, texture enhancers, and nutraceuticals) applied on different minimally processed fruits and vegetables, highlighting the coating ingredients, the application methods and the effects on food shelf life and quality.

## 1. Introduction

During storage, food is subjected to a process of quality degradation, this phenomenon being a major problem faced by food producers, contributing significantly to food waste. In the last years, novel and smart food processing technologies (UHP—Ultra High Pressure, PEF—Pulsed Electric Field, MAP—Modified Atmosphere Packaging, RF—Radio Frequency, Active Packaging, and others) were developed with the aim of contributing to food preservation extension, shelf life-prolonging, and, consequently, food waste reduction [1,2,3,4]. However, not all of these novel technologies represent a real solution in the market due to their impact on consumers’ attitudes [5,6].

In the last decades, the food products consumer market developed some changes in terms of sustainability and health implications of food processing and packaging [7]. Moreover, consumers are looking for less processed products or minimally processed products which have a convenient preservation period, are healthy, and present great nutritional value. These requirements are more stringent when discussing highly perishable food products, such as fruits and vegetables. This increasing demand is now a real challenge to the food producers in order to develop pertinent and sustainable preservation techniques [8].

A novel way to diminish this problem is the use of edible packaging, edible coatings, or edible films, which can provide an additional protective layer(s) for fresh products, thus increasing their shelf life by delaying microbial spoilage and providing moisture and gas barrier properties [9]. 

Nowadays, consumers prefer food less processed and healthier food products and so the research activity on edible packaging systems is rising every year. Edible films and coatings are designed as a primary packaging material for foodstuffs having edible components and so as to help to maintain sensorial properties such as aroma, taste, and appearance. Fruits and vegetables that are coated with edible films have longer shelf lives and their ripening processes are delayed [7,10]. 

Edible coatings have been used since the 12th and 13th centuries in China, where a thin coat of wax was applied on orange fruits. In the 15th century, it was discovered that Japan designed an edible coating material made from boiled soybeans, which was applied on different food products in order to improve their appearance [11]. 

In recent years, the market share of edible packaging has seen an increase, being valued at $697 million in 2016, and by 2023 it is expected to grow to $1097 million [12]. There are two distinct ways in which edible packaging can be used in the food industry. Edible coatings can be applied directly on the food product or can be wrapped around the food product in the form of a preformed film [13].

Although edible coatings and films can help prolong the shelf life of different food products, the food industry faces the challenge of consumer acceptance towards novel processing techniques [14]. Understanding how consumers form and perceive attitudes in relation to new technologies and products is important for food chain innovation, since consumer acceptance is crucial to the development of successful food products [1,15]. Several studies regarding consumer acceptance towards novel processing technologies and techniques have been made, i.e., nanotechnology [16], radio frequency [6] food irradiation [17], and edible films and coatings [18,19]. This review focuses on the latest research on edible formulation applied on different minimally processed fruits and vegetables, highlighting the technological aspects such as coating ingredients and formulation, application methods, and the effect on food shelf life and quality, including nutritional quality. Edible films and coatings, their composition, and methods of application as well as the functional additives in edible coatings for minimally processed fruits and vegetables are presented.

## 2. Methods

The articles included in the present review were gathered and synthesized from online publisher databases. In order for the articles to be included, they were screened for their data and presentation to be correct and clearly described. Selected articles were those that were published after 2010, and that referred only to the coatings of fruits and vegetables. In addition, articles were excluded if their data and results did not take into consideration the effect on the nutritional values of the coated samples and if the coating methods, treated food products, or the effects of the treatments were not clearly stated. All the selected articles presented a shelf life assessment. The content analysis of the reviewed studies was aimed at defining the scope of analysis, evaluating the content, and, in the end, classifying the content in several directions such as: highlighting the importance of functional coatings and edible coatings; highlighting the food product groups which have been treated with functional coatings or edible films and the results of these treatments; and highlighting the film or coating matrix and the functional compound (antioxidants, antimicrobials, emulsifiers, anti-browning agents, flavoring agents, colorants, and probiotics). The literature search included research articles and reviews on the following topics: “application of food coatings”, “functional coatings”, “edible films”, “edible coatings for fruit preservation”, and “fruit and vegetable edible coatings”. A total of 87 research articles published in the last decade were selected and synthetized from publisher databases. The vast majority of the research studies that related to functional coatings and edible films presented studies on fresh and minimally processed fruits and less on vegetable products, which are coated with different formulations of functional coatings or edible films. The studies used in this review were screened by two separate reviewers and each made a report containing the most important facts found in the article. No automated tools were used in the process.

## 3. Composition and Methods of Application of Edible Coatings

An edible coating is generally defined as a coating layer made from chemical or biological materials which are applied as a thin layer or layers on the product surface in order to prevent gaseous exchange, thus retarding the ripening process. Another definition for edible coatings was given by Baldwin et al. (2011) [20], defining them as a thin comestible layer that can be applied to the fruit’s surface in order to create a barrier between the fruit and the environment. Jongsri et al. (2016) [21] stated that edible coatings must provide a partial barrier to water movement, so moisture loss can be reduced, and, at the same time, can modify the atmosphere around the fruit by acting as a barrier to gas exchange. 

The main components used for edible coatings and films are lipids, polysaccharides, and proteins, but other materials need to be used too, such as resins, solvents, and plasticizers, in order to obtain different characteristics for the edible coatings. The flexibility and permeability of edible coatings is conferred by the use of plasticizers, tensile strength by the use of solvents, and water vapor permeability prevention is obtained with the help of resins [10,22].

Yifan et al. (2020) [23] developed self-healing coatings based on sodium alginate (SA) and l-menthol-beta-cyclodextrin-graft-chitosan for the improvement of the postharvest quality of fruits and vegetables. The developed materials presented good characteristics, showing that the addition of l-menthol led to dense, smooth, and transparent coatings with better mechanical and self-healing characteristics. Fu et al. (2021) [24] developed edible films based on waste fish scale-derived gelatin, chitosan, and CaCO_3_ nanoparticles. The edible films presented important characteristics such as UV absorption, antimicrobial activity, great mechanical properties, and non-toxicity. Furthermore, the developed edible films were hydrophilic, which means that they can be easily removed from fruit by washing. Fan et al. (2021) [25] developed an edible composite film (PAX) based on pectin, sodium alginate (SA), and xanthan gum (XG). The film showed important properties, such as a tensile strength which could reach a maximum value of 29.65 MPa at a concentration of 4 g/L XG, 18 g/L glycerol, and 20 g/L CaCl_2_. In addition, the elongation at break was of 19.02% and the water vapor transmission rate was of 18.12 × 10^−11^ g/(m^2^·s·pa). Active films based on chitosan and gum arabic also containing cinnamon essential oil were developed by Xu et al. (2019) [26]. The analysis performed on the developed films showed that there were electrostatic interactions between chitosan and gum arabic, forming an entangled structure. In addition, the addition of gum arabic enhanced the water barrier properties and the antioxidant effectiveness of films.

The developed edible films and coatings could also meet environmental concerns due to the fact that they are generally obtained using biocompatible, biodegradable, low-toxicity, and GRAS (Generally Recognized As Safe) materials [27].

After the production of these edible coatings, they need to be applied to the desired food products. This can be done by different methods like brushing, spraying, dipping, extrusion, a fluidized bed, panning, and solvent casting. Both the food products and the edible coatings need to be examined and tested before the best method of applying the edible coating is selected. Carbon dioxide or oxygen permeability, high or low water vapor permeability, and good mechanical resistance are only some of the characteristics that need to be assessed for the coating materials [11,28]. 

On commercial use, extrusion and spraying processes are desired methods for food product coatings or film formation. At a laboratory scale, coating and film formation are obtained by dipping and casting processes. Despite of the existence of several methods in which the edible films can be applied, the most used ones are casting (a wet process) and extrusion (a dry process) [13].

## 4. Edible Films and Coatings for Fruits and Vegetables Preservation

Fruits and vegetables are unanimously recognized as the main sources of vitamins, minerals, antioxidants, and fiber in the diet of consumers. At the same time, their short shelf life is well known, due to their high moisture content (75–95%), which is the main cause of their rapid degradation. The process of decay of fruits and vegetables is well explained by their nature, living products that go through the natural process of maturation, aging, and degradation. 

Fruits are divided into two groups according to the mechanisms underlying the ripening process. From this point of view, they are divided into climacteric and non-climacteric fruits. While fruits belonging to the second category do not continue the ripening process after being harvested, those in the first category can ripen even after harvest and produce more ethylene than non-climacteric ones, so they are more susceptible to spoilage caused by microorganisms. For this reason, suitable packaging and coating technologies are more than necessary for a reasonable shelf life of these food products [29].

Different types of nanocomposite films and edible coatings are used nowadays extensively on fresh fruits and vegetables for the prolonging of their shelf life using the same principles as modified atmosphere methods, which already shown good results for preserving fruits and vegetables quality [29,30]. 

The mechanisms of action of these edible films are based on the formation of the semipermeable safety barrier around the vegetables and fruit, reducing considerably the loss in quality attributes. These properties are well-preserved by the relatively high number of biopolymers such as starch, pectin, carrageenan, alginate, chitosan, and xanthan gum, which have been extensively used to create edible films and food coatings [8]. 

Numerous studies have already proved the positive effect of chitosan and alginate-based edible coatings on the quality preservation of fruits and vegetables. These coatings ensure a substantial barrier against water release and behave as micro-environments ensuring an optimal concentration of gases and leading to delays in ripening, which is equivalent to a longer preservation period [8]. Chitosan, functionalized chitosan, and chitosan nano-formulations are excellent candidates for creating edible coating for fruit products such as cut pineapple [31], cut cherry tomatoes [32], apricots [33], peaches [34], blueberries [35], etc. Furthermore, a study made by Sun et al. (2021) [36] compared the shelf life effect of three bio-based nanomaterial coatings from chitin, cellulose, and chitosan that were applied on kiwi, avocado, strawberry, banana, nectarine, and apricot. Both wood nanocrystals as well as chitosan nanofibers presented the best antifungal activity. Moreover, color changes and weight loss of the coated strawberries decreased by half compared to the control samples. Jongsri et al. (2016) [21] studied the effect of coating ’Nam Dok Mai’ mango fruits (a tropical fruit from the drupe family), a highly perishable fruit after harvesting, with different chitosan solutions (high molecular weight, medium molecular weight, and low molecular weight). The samples were stored for 16 days at a room temperature of 25 °C. The results showed that the high molecular weight chitosan-based coating led to a delayed ripening of the treated samples, which presented the highest value of titratable acidity, of fruit firmness, and also of the reduction in weight loss, ethylene production, and respiration rate. In addition, the study showed that the mango fruits coated with chitosan with high molecular weight managed to maintain their ascorbic acid and DPPH value over the storage period. It can be stated that the coated mango samples had a better quality compared to the control and the other samples, and their shelf life was significantly improved.

Fresh-cut apple slices were coated firstly with a bilayer probiotic with *Lactobacillus plantarum* 299v containing a carboxymethyl cellulose solution edible coating and, after, as a second coating with zein. The apple slices were stored for a period of 7 days at a temperature of 4 °C, and in this time *L. plantarum* 299v remained stable (>6 log CFU/g). The coated samples showed reduced weight loss and microbial growth—an inhibition in the proliferation of spiked *Listeria monocytogenes* during storage [37].

Blackberries stored at 40 °C for 20 days were coated with an edible and biodegradable film based on starch and nystose in order to prolong the shelf life of the coated fruits. The tests were comprised of three types of samples—control fruits, samples coated with starch, and samples coated with edible starch-nystose coating. It was shown that the fruit samples coated with starch and starch-nystose had positive effects in delaying the increase in pH and maintaining firmness and anthocyanin content. The sensory analysis data revealed that the coated samples presented the same interest for buyers as well as good acceptance without major visual modifications compared to the control samples [38].

The enhancement of the fruit’s exterior appearance and the decrease in fungal development is possible because a slowing down of respiration and senescence without causing anaerobiosis is present when the edible coatings are applied [29,39]. 

Edible coatings have already been tested by a large number of researchers and have been applied over a large variety of fruits with the principal aim of extending their shelf life and maintaining their biochemical properties. For example, a very large number of studies were made on fruits like berries and tropical fruits [29], papaya [40,41], goji [42], oranges [43,44,45], cherry [46], mango [47,48], strawberries [49,50], and also on vegetables like tomatoes [29,51,52,53], carrots [54], and broccoli [19,55]. 

Chitosan-based edible coating with cellulose nanofibers and curcumin have been reported to maintain the shelf life and quality attributes of kiwifruits, such as: lower loss in mass and firmness, slower respiration rate, and the reduction of microbial growth [56]. 

The common results of these studies showed that whole fruits and vegetables samples coated or treated with edible coatings presented an improved shelf life (by decreasing respiration rate and weight loss) and better physical-chemical attributes (like firmness, titratable acidity, and vitamin C content). As for fresh-cut fruits, the most benefits are reducing water loss, increasing soluble solid content (SSC), and maintaining the color of the products.

## 5. Edible Films and Coatings with Functional Additives for Minimally Processed Fruit Application

The next steps in this field are strongly represented by research focused on improving the effect of edible films based on a polysaccharide matrix by adding functional ingredients such as [57]:

- *plasticizers* (glycerol, sorbitol, sucrose, mannitol, acetylated, monoglyceride, polyethylene glycol, and xylitol) added to coatings to increase flexibility and prevent coatings from blistering, flaking, and cracking;
- *emulsifiers* (soy lecithin, stearic acid, and Tweens) and *surfactants* (Tweens) added to improve coating adhesion;
- *antimicrobial agents* (nisin, natamycin, phenolic compounds, natural seed extracts, and essential oils—like cinnamaldehyde, eugenol) added to improve the antimicrobial activity of a coating;
- *antioxidants* (ascorbic acid, citric acid and α-tocopherol) added to coating matrices to prevent oxidative rancidity, degradation, and discoloration;
- *nano-compounds* (like metal oxides as ZnO or TiO_2_).

These functional compounds are seen now as a key component of edible films/coatings for prolonging the shelf life of fruits and vegetables and testing has already started for various fruits (guava, pear, and blueberries) and vegetables (cucumber, capsicum, and mushroom) focusing also on safety and nutritional aspects [8].

Leena et al. (2020) [58] obtained an effective delivery system using nano-structured edible coating based on zein enriched with resveratrol with the possibility of using a controlled release system. An electrospinning process was used in order to encapsulate the resveratrol (in concentrations by 2%, 5% and 10%) in the zein nanofibers. The obtained edible coating by electrospinning of resveratrol loaded zein nanofibers was applied on apple slices. The study showed that the coated apple slices retained better color, due to the antioxidant effect of resveratrol added as a functional ingredient, and the control of moisture loss also improved.

Arnon-Rips et al. (2021) [59] conducted a study for obtaining a new structure of edible coatings using a covalent linkage mechanism. Two functional compounds (vanillin and trans-cinnamaldehyde) were bound to chitosan (polysaccharide matrix) by Schiff base reaction and reductive amination. The functionalized structure of chitosan was analyzed and tested as an edible coating in the case of fresh-cut melon samples. The results of the study showed that the tested films produced well-adhered coatings that managed to increase the fresh-cut melon quality and shelf life without altering the sensorial attributes. In order to test the antibacterial effect, mandarin juice was added to the chitosan and vanillin and trans-cinnamaldehyde mixture, and the results showed a 6 log CFU/mL microbial count reduction, which clearly demonstrates this effect. 

Fresh-cut apple samples were coated with edible coatings (carboxymethyl cellulose and Aloe Vera) and anti-browning agents in different combinations, with only one active ingredient or both. The treated samples were packed in polypropylene trays and stored at 5 ± 2 °C. Multiple parameters were studied along the storage period of the samples, such as: physical properties (color, physiological loss in weight, and firmness), bio-chemical properties (ascorbic acid, total antioxidant, phenol, polyphenol oxidase, and peroxidase enzymes) and microbiological indicators. The samples coated with the edible coating material along with the anti-browning agents helped preserve the quality of the samples. As for the microbiological assay, it was observed that apple slices coated with carboxymethyl cellulose and Aloe Vera had a significantly lower microbial load. The coated apple samples showed an improved firmness compared to the untreated samples. Polyphenol oxidase and peroxidase enzyme activity was also lower in the coated samples [60].

Alginate-based edible coatings enriched with Aloe Vera were developed using the Box-Behnken design in order to optimize the minimum water vapor permeability. In order to create these films, titanium oxide nanoparticles (nTiO_2_) were incorporated in different percentages within the film. Mechanical and antimicrobial properties were improved after the incorporation of titanium oxide nanoparticles. Tomato samples were treated with these Aloe Vera and alginate-based edible coatings and shelf life studies showed significant resistance to weight loss and spoilage when alginate/Aloe Vera film containing 5 wt% of nTiO_2_ was applied [61].

Salas-Méndez et al. (2019) [53] investigated the effects of nanolaminate coatings incorporated with extracts of *Flourensia cernua* on tomato in order to extend their shelf life. The nanolaminate coating was made from polyelectrolyte solutions of alginate and chitosan that had been treated with ethanol extracts of *Flourensia cernua*. The samples were coated with this material and several parameters were tested: physicochemical analyses, ethylene production, and microbial growth. The treated samples presented weight loss and microbial growth reduction. In addition, ethylene production was slower, and the tomato firmness and color were better preserved. This shows that the nanolaminate edible coatings could improve the shelf life of tomato samples.

Lara et al. (2020) [62] studied the effect of spray-coating of fresh-cut lotus roots with xanthan gum-based edible coatings. In order to have multiple variants, the study tested several variants of edible coating solutions, consisting in three concentrations of xanthan gum solutions (0.1%, 0.3%, and 0.5%). In all of the above solutions 2% (*w*/*w*) citric acid was added as an anti-browning agent and 1% (*w*/*w*) glycerol as a plasticizer. Fresh-cut lotus roots were then sprayed with these solutions in a 5 mm thick layer for 20 s and stored at a temperature of 5 °C for 16 days in polyethylene bags. Morphology, pH, color, and microbiological determinations were performed, and it was observed that the treated samples had a significant reduction in the total color changes compared to control samples which were not sprayed. In addition, the enzymatic browning of fresh-cut lotus root during storage was decreased. A lower microbial count was recorded to the treated samples compared to non-coated fresh-cut lotus root samples in terms of Bacillus subtilis growth rate in the first 24 h of storage.

Due to the long food chain characteristics of bananas, scientists are looking for more sustainable methods for preserving them. Alali et al. (2018) [63] studied the effects of gum arabic (GA), salicylic acid (SA) and their mixture in the form of coatings on the quality of ‘Grand Nain’ bananas during postharvest storage. Nutritional value (total phenols, flavonoids, and vitamin C) showed a good response in the case of GA application and less favorable responses in the case of SA application. In addition, the peel browning index was better in the case of the GA coating. According to Sinha et al. (2021) [64] pear samples coated with chitosan-enriched 2.0% and 2.0 mM salicylic acid stalled the development of internal browning throughout the storage period.

Basiak et al. (2019) [65] studied the effects of coating plums with two different starch-based edible coatings, one containing only starch and the other one containing starch and whey protein. The effects of the coating materials applied on the surface of plums on water loss were determined by studying resistances in the water vapor pathway. The dynamic behavior of two starch-based coatings both at high and low potential water losses was evaluated in the experiments. The results showed that when applying the coatings in a three-layered model, the starch and starch-whey protein coatings increased the total resistance in the water vapor pathway of individual plums by 60–75% at high transpiration potentials. An increase of 11–20% was observed at lower transpiration potentials.

Arabic gum, xanthan gum with lemon grass essential oil 1% *w*/*v* and carrageenan edible coatings were studied by Wani et al. (2021) [66]. Postharvest quality tests were made over strawberries samples treated with the three developed edible coatings, over a period of 12 days. The result showed that the coated strawberry samples had a reduction in weight loss, retained the ascorbic acid better, had better antioxidant activity, and had improved firmness. The edible films with carrageenan gum managed to retain the anthocyanins levels and phenolic compounds during the storage period. In addition, the best results in terms of maintaining quality during storage were the coatings containing carrageenan gum.

A study carried out by Muley et al. (2020) [67] investigated the effects on the shelf life extension of strawberries with a novel functional coating made from whey protein isolate, chitosan, and glycerol. The obtained films were cast at temperatures over 60 °C, and several physical analyses were performed (antioxidant activity, color, oxygen transfer rate, crystallinity, etc.). Fresh strawberry samples were coated with these films and put into storage at two different temperatures (5 °C and 20 °C) for 8 days. A series of biochemical and physical analyses of the samples was performed in order to assess their shelf life. The results showed that the coated samples had a reduction in weight loss, pH, color modifications, titratable acidity, total phenolics, and DPPH. In terms of shelf life extension, the shelf life was extended from 3 to 5 days for the untreated samples and 5 to 8 days for the coated samples.

Alejandra Moreno et al. (2020) [68] studied the shelf life of raspberries (*Rubus idaeus* L.) that were coated with an edible coating based on gelatin with ethanolic-based extract of propolis (PEE). Several antifungal activity tests were performed for the propolis extract (PEE) over a series of major fungal spoilage of fruits and vegetables (*P. digitatum*, *P. expansum*, *P. italicum*, *A. alternata*, *A. carbonarius,* and *B. cinerea*). The PEE was incorporated in the gelatin edible films by two mechanisms: (1) mixing PEE directly in the matrix of the protein and (2) by encapsulation of the PPE in the zein, forming nanocapsules which were than incorporated. The incorporation of PEE changes the mechanical properties of the edible films which become more flexible and deformable, but also more colored films with lower transparency. The edible films were then applied to the raspberries samples and the fungal decay was assessed at cold storage at 5 °C. A notable antifungal activity against the tested fungus was observed over the treated samples, showing a greater inhibitory effect on *P. digitatum* and *B. cinerea* strains. Using the coating film with PEE added enhanced the shelf life of raspberries during cold storage.

A bioactive binary blend film made of pectin, pullulan with *Vitis vinifera* grapeseed extract was used to prolong the shelf life of peanut samples [69]. A dense film structure with food mechanical strength was obtained and applied as an edible coating over the tested peanut samples and then stored for a period of 30 days at 20 °C. The results showed that the coated samples had a better shelf life by reducing lipid oxidation, thus delaying rancidity. In addition, the film made of pectin, pullulan and *Vitis vinifera* grapeseed exhibited antibacterial activity against *E. coli* and *L. monocytogenes*.

Kumar et al. (2020) [70] investigated the effect of some newly developed composite edible coatings from chitosan-pullulan (50:50) and peel extract from pomegranate, on the shelf life and quality parameters of green bell pepper samples. The tests were carried out over a period of 18 days at two different conditions: (i) at room temperature 23 ± 3 °C, and RH: 40–45%, and (ii) at chilling temperature—4 ± 3 °C, and RH: 90–95%. The new formulation of edible coatings maintained total soluble solids content, titratable acidity, pH, phenolic content, flavonoid content, antioxidant activity, firmness, and sensorial attributes over the entire storage period. In addition, the coatings helped to significantly reduce physiological loss in weight and color browning. It can be stated that the treatment with the newly developed edible coatings had a positive effect on the quality and shelf life of the samples, in both tested conditions. 

Vilaplana et al. (2020) [71] studied the antifungal effect of an edible coating based on chitosan over blackberry samples, which have a short shelf life because of the microbial load. The actual tests were made by using different formulations of the developed chitosan edible coating with acetic or lactic acid and then applied on the blackberry samples in order to evaluate the antifungal activity over *Mucor racemosus*. The treated samples were kept at refrigeration temperatures (4 °C) for a period of 14 days. In this study samples coated with the edible coating, samples treated with chemical fungicide imazalil (0.4 g L^−1^), and untreated samples were compared. The results showed that the samples coated with chitosan and lactic acid had the best antifungal effect over *Mucor racemosus*. 

A new edible functionalized coating system, based on carboxymethyl cellulose (CMC) and *Lactobacillus plantarum* in different concentrations, was developed and applied on fresh strawberries in order to study the impact on their shelf life at 4 °C as well as on their physicochemical and microbiological characteristics. As *Lactobacillus plantarum* is a probiotic, the treated strawberry samples helped to reduce the growth rate of molds and yeasts on the surface of strawberries compared to control samples, due to competitive and antimicrobial properties of *Lactobacillus plantarum*. In addition, the study showed that the number of viable *Lactobacillus plantarum* strains in all treatments was constantly higher than 6 log CFU g^−1^ for all tested periods and it increased by inoculation of a higher amount of *Lactobacillus plantarum* strains in the coating solution, which is a positive aspect for the functional role of the proposed edible coating [72].

Vishwasrao & Ananthanarayan (2017) [73] studied the shelf life extension effect of treating sapota fruits (a large berry) with an edible coating based on a mixture of methyl cellulose (MC) and palm oil (PO). The results showed that the tested fruit samples had a reduced peroxidase, polyphenol oxidase, and pectin methylesterase activity during postharvest ripening. As for the physiological properties, it was observed that the edible coating that was used had a good effect on anti-browning and discoloration, ascorbic acid retention, and delayed loss of total phenolic content of the tested samples. Overall, the edible coating made from methyl cellulose (MC) and palm oil (PO) managed to maintain the treated sapota fruit samples, extending the shelf life by three days at 24 ± 1 °C and 65 ± 5% RH compared to the control samples.

In the study performed by Mendy et al. (2019) [41] papaya fruits (large berry) were coated with aloe vera-based gel, and the shelf life and quality were assessed in order to outline the edible coatings’ effects. The coated papaya samples were stored for 15 days and different analyses such as higher soluble solid concentration-SSC, pH, titratable acidity-TA, ascorbic acid-AA, total carotenoids content, total phenolic content-TPC, total flavonoids content-TFC, and DPPH scavenging activity were performed at 3 days each. At 50% gel concentration, the papaya samples coated with two types of edible coatings exhibited a diminished microbial growth rate after 15 days. As for the quality parameters, the coated samples presented a higher soluble solid concentration, titratable acidity, ascorbic acid, total carotenoids content, total phenolic content, and total flavonoids content compared to the uncoated samples. It can be stated after the analysis of these results that the shelf life of the coated papaya samples was improved by 25%. Moreover, Passafiume et al. (2020) [74] studied the effect of three edible coatings based on aloe vera gel, hydroxypropyl methylcellulose, and lemon essential oil-based composite coating on prolonging the shelf life and quality of fresh-cut Hayward kiwis. The treated samples were stored for 10 days at refrigeration temperatures and a series of microbiological, chemical, and sensory tests were performed at days 2, 4, 7, and 10. The edible coatings were applied by spraying method. The results showed that the coated samples presented reduced weight loss and browning and maintained higher firmness, brightness, greenness, and soluble solid contents (SSC). Aloe vera gel and aloe vera gel + lemon essential oil reduced the microbial load compared to the control samples.

Khorram et al. (2017) [75] studied the effect of edible coatings, obtained from gelatin (5, 6, and 7%), Persian gum (3.5, 4, and 4.5%) and 9, 10, and 11% shellac, on ‘Valencia’ oranges (which are considered a hesperidium, a kind of modified berry [76]) during storage. The composition of the edible samples was established based on the properties of the selected ingredients, which are easy to dissolve and inexpensive. The coated oranges were then stored for 60 days at a refrigeration temperature of 5 °C. Sample evaluation was made every 20 days and images of the coated surfaces were obtained by scanning electron microscopy. The coated samples were compared with uncoated ones or with wax-coated fruits. In order to test the capabilities of the edible coatings obtained, physiological and physicochemical analyses were performed: total phenolic content, total antioxidant capacity, titratable acidity, pH, ascorbic acid content, total soluble solids, respiration rate, weight loss, fruit firmness, and total soluble solids. The titratable acidity and acid content decreased as the storage time was increased, but the pH, total phenolic content, and total antioxidant capacity values increased. In all coated samples, a glossiness effect was observed. However, fruit coated by gelatin and Persian gum coatings, showed visible cracks with increasing storage time. From all edible coating variants, the shellac was the best, because it formed a non-sticky and odorless coating with a high glossy aspect through the entire tested storage period. 

Jing-Fan et al. (2019) [42] investigated the effect of blend coatings with lotus leaf extract (LLE) on the quality of fresh goji berries postharvest stored at ambient temperature. The best results were obtained using 0.2% LLE, 1% basic coating (sodium alginate, konjae glucomannan, and starch in proportion of 2:3:3), 1% glycerin and 0.5% CaCl_2_. The results show that LLE incorporated coatings extend the shelf life of goji berries for about 4 days and had a positive effect on the maintenance of their quality. 

In addition to increased perishability of strawberries due to a delicate tissue and a high sugar content, the transport conditions of these berries is an important additional factor in the reduction of their shelf life. A much-focused study on this issue was performed by Dhital et al. (2017) [49] trying to find a protective solution by using the edible coating of ‘Chandler’ strawberries subjected to simulated vibrations of local transportation. In this sense, six types of coatings were developed and evaluated based on the quality of coated berries. The coatings were based on incorporation of different proportions of curcumin and limonene, as natural antimicrobials, and their liposomes in methyl cellulose. The first conclusion was that the protection of the vibrated samples, which had a lower shelf life than non-vibrated samples, indicates a need for a more robust coating in order to remain intact during road vibrations. Regarding the shelf life and quality of the treated samples, it was clearly demonstrated that limonene liposomes showed significantly lower fungal growth compared to the control on the 14th day of storage and the titratable acidity and total phenolic contents were also higher in limonene-coated strawberries compared to other coatings. Future research is planned to improve integrity of the coatings, testing liposome coatings of limonene with different particle size. 

Guerreiro et al. (2015) [77], performed a complex study focused on the testing of different sodium alginate (AL)-based edible coatings, enriched with essential oils (Eugenol and Citral) as antimicrobial compounds, for maintaining postharvest quality and prolonging storage life of *Arbutus unedo* fresh fruit (red berry). The quality monitoring (through physicochemical and biochemical analyses—color, firmness, weight loss, antioxidant capacity, microbial growth, and taste panels) of the coated arbutus fruits, showed good preservation in the case of using the coatings with formulation of AL 1% + Eug 0.20% and AL 1% + Cit 0.15% + Eug 0.10%. In a study performed by Oyom et al. (2022) [78] an edible coating formulation containing modified starch from sweet potatoes and cumin essential oil was applied on pear samples (*Pyrus bretchneideri* Rehd.). The coating method used in the tests was dipping the sanitized and disinfected samples into the coating formulations, and the coated samples were stored for 28 days at a temperature of 25 °C. The coating treatment managed to suppress the respiration rate and delay the weight loss and maintained flesh firmness after 21 days, due to the adhesiveness of the modified starch coating which formed a thin layer on the pear and maintained the pericarp and freshness. The antibacterial and antibiofilm effect of newly developed edible nano-emulsion coatings containing sodium alginate and essential oil extracted from sweet orange was tested in a study performed by Das et al. (2020) [79]. Two pathogenic bacterial strains were selected in this study, *Salmonella* and *Listeria*. Beside the antibacterial and antibiofilm effect, the quality properties of tomato samples were analyzed. The samples were stored at a temperature of 22 ± 2 °C, over a 15-day storage period. The samples coated with the edible nano-emulsion coatings did not show bacterial growth of the two tested microorganisms. In terms of the quality characteristics of the coated tomato samples, it was shown that the firmness was significantly enhanced, up to 33%, and a decrease in weight loss up to 3-fold lower than uncoated samples. 

The incorporation of pomegranate seed oil in edible coatings of *Chlorella* sp. have positive effects on conservation and delay ripening of *Spondias tuberosa*, prolonging the shelf life of fruits and keeping them firmer, greener, and turgid. In addition, the coating with 2.0% of *Chlorella* sp. associated with pomegranate seed oil under cold storage at 14 ± 2 °C and 85 ± 5% RH for 12 days maintains fruit quality such as vitamin C and phenolic compounds [80].

In another study, fresh-cut pineapple fruits were coated with sodium alginate containing different concentrations of citral nanoemulsion (0.1%, 0.5%, and 1%) and stored for 12 days at 4 °C and a relative humidity of 90%. The edible coating containing 0.5% and 1% citral nanoemulsion improved the physicochemical attributes (better color retention, low respiration rate) as well as reduced microbial growth [81].

The effects of newly developed hydroxyethyl cellulose and sodium alginate edible coating with asparagus waste extract was tested on the postharvest quality of strawberries [82]. Three different samples of the developed edible coatings were developed, each with different concentrations of asparagus extract (1.5 g/100 mL hydroxyethyl cellulose coatings, 1.5 g/100 mL sodium alginate coatings and 1.0 g/100 mL hydroxyethyl cellulose/0.5 g/100 mL sodium alginate). The edible coating containing 1.0 g/100 mL hydroxyethyl cellulose/0.5 g/100 mL sodium alginate managed to maintain the total flavonoid and phenolic contents as well as significantly delayed color change and weight loss. In addition, this edible coating presented an antimicrobial effect on *Penicillium italicum*. Duong et al. (2022) [83] developed an alginate-based edible coating material in order to maintain the shelf life of rose apple fruits during cold storage. Various concentrations of CaCl_2_ solutions were mixed with sodium alginate in order to find the best formulation by determining the barrier and mechanical proprieties of the films as well as performing physiochemical analyses of the coated apple samples. The addition of the CaCl_2_ solutions decreased water vapor permeability and oxygen permeability compared to the alginate films. As for the physiochemical results, the coated samples presented a significantly reduced respiration rate and weight loss as well as a delayed chilling injury. The total phenolic content and antioxidant activity in fruits coated with the newly developed coatings were 1.46- and 1.48-fold higher than those of the control, over storage at refrigeration temperature for 10 days.

Starting from the antimicrobial effect of blueberry (*Vaccinium* spp.) fruit and leaf extracts (BLE), Yang et al. (2014) [84] tested the effect of the chitosan-based coatings with different concentrations of BLE on the quality of fresh blueberry fruits in different postharvest storage conditions (at 2 ± 1 °C, 95 ± 2% relative humidity (RH) for 35 days; at room conditions for 3 days). The results regarding the use of the chitosan-based coatings and BLE are promising for extension of shelf life in the conditions of maintaining the nutritional quality of fresh blueberries during postharvest storage. The 2% chitosan coating with 8% or 12% BLE showed a shelf life extension compared with the control, and the coating with BLE plus MAP had a more effective control of fruit decay.

A synthesis of best results is presented in Table 1, and these results confirm that the coating technology for fruits and vegetables post harvesting preservation can be considered a sustainable solution, with matrices based on natural and renewable materials which are also biodegradable. 

## 6. Conclusions

The current review presents a recent investigation of newly developed coatings applied on fresh and minimally processed fruits and vegetables. The latest developments in this field are represented by the extensive use of chitosan or alginate as the main component of the edible coatings to which different functional ingredients are added, like EOs, nano-forms, antioxidants extracts, and probiotics. The role of these functional ingredients is not limited to the basic function of the protective layer, which assures extending shelf life, but also provide functional properties, such as antioxidant capacity, probiotic properties, better sensory attributes, and higher vitamin C content, having a positive impact on consumers. A better understanding of the mechanism of the edible functional coatings and their promotion among consumers could help to extend their application in fruit and vegetable preservation. Moreover, the edible coatings and film can become a very promising method that could be applied for delivering bioactive compounds in order to increase bioavailability. Despite the various and clear research in this field and due to very specific coating technology applied for different fruit and/or vegetables, with different proposed aims (starting from improving shelf life to preserving a high nutritional value or increasing certain nutritional features of the products) the subject is of permanent topicality and experiments are absolutely necessary starting from the known data.

## Figures and Tables

**Table 1:** Effect of edible coatings/films with different functional ingredients on fruits and vegetables quality.

Film/Coating Matrix (Coating Method)	Functional Compound (Role)	Coated Fruits or Vegetables	Advantages of Coating Technology and Main Results of Study	Reference
*Polysaccharides and their derivatives-based matrix (starch and its derivatives, cellulose and its derivatives, alginate, pectin, chitosan, and gums)*				
Methyl cellulose (MC) (Dip coating)	Palm Oil (PO) (anti-browning agents, antioxidants, and antimicrobials)	Sapota fruits (a large berry)	Decrease PO, PPO, PME activity and discoloration; Increase anti-browning effect and retention of ascorbic acid; Delay the loss of total phenolic content; Extend the shelf life by three days	[73]
Methyl cellulose (MC) (Dip coating)	Curcumin; Limonene (antioxidants, antimicrobials)	‘Chandler’ strawberries	Decrease fungal growth; Increase TPC, TA	[49]
Carboxymethyl cellulose (CMC) (Dip coating)	Aloe vera (anti-browning agents, antioxidants, and antimicrobials)	Apple slices	Decrease PO and PPO activity Lower microbial load; Better firmness; Anti browning effect.	[60]
Carboxymethyl cellulose (CMC) (Coating)	*Lactobacillus plantarum* (antimicrobials, probiotic)	Strawberries	Reduce the growth rate of molds and yeasts on the surface of strawberries; Improve functionality (as a probiotic)	[72]
Hydroxyethyl cellulose and sodium alginate (Dip coating)	Asparagus waste extract (antioxidants, antimicrobials)	Strawberries	Maintain the TFC and TPC, delay color change and weight loss	[82]
Hydroxypropyl methyl cellulose (Spraying)	Aloe vera gel and lemon essential oil (antioxidants, antimicrobials)	Hayward kiwis	Reduce weight loss and browning, maintain higher firmness, brightness, greenness, and TSS Reduce the microbial load	[74]
Chitosan solutions with different molecular weight (Dip coating)	Chitosan (antimicrobials)	’Nam Dok Mai’ mango fruits	Delay ripening; Increase TA, Fruit firmness, Reduction of weight loss, ethylene production, and respiration rate; Maintain the ascorbic acid and AOC (the case of chitosan with high molecular weight)	[22]
Chitosan (Dip coating)	8% and 12% blueberry (*Vaccinium* spp.) fruit and leaf extracts (BLE) (antioxidants, antimicrobials)	Blueberries (*Vaccinium* spp.)	Decrease microbial growth and decay rate; Increase shelf life	[84]
Chitosan (Dip coating)	Acetic or Lactic acid (antimicrobials)	Blackberry	Antifungal effect over *Mucor racemosus*	[71]
Chitosan (Coating)	Vanillin and trans-cinnamaldehyde and mandarin extract (antioxidants, antimicrobials)	Fresh-cut melon	Reduce microbial load; Increase storage life; Maintain sensorial attributes	[59]
Chitosan-pullulan (Dip coating)	Pomegranate peel extract (anti-browning agents, antioxidants, and antimicrobials)	Green bell pepper	Decrease weight loss and color browning; Maintain firmness, TPC, TFC, AOC, and sensorial attributes	[70]
Chitosan and cellulose nanofibers (Dip coating)	Iron particles, curcumin (antimicrobials)	Kiwifruits	Reduce weight loss and firmness and reduce respiration rate	[56]
Chitosan and glycerol (Coating)	Whey protein isolate (antioxidants, antimicrobials)	Strawberries	Decrease weight loss, pH, color modifications, TA, TPC, and DPPH; Extend shelf life with 60%	[67]
Chitosan, Alginate (Coating)	*Flourensia cernua* ethanol extract (antimicrobials)	Tomatoes	Decrease weight loss; Decrease microbial growth and ethylene production; Maintain firmness and color	[53]
Chitin, cellulose, and chitosan (Coating)	Chitosan (antimicrobials)	Strawberries	Decrease microbial growth, decrease color changes and weight loss	[36]
Chitosan (Coating)	Salicylic acid (antimicrobials)	Pears	Decrease PPO activity; Stalled the development of internal browning throughout the storage period	[64]
Chitosan (0.05%) (Coating)	Cinnamon essential oil (0.1%), trans-cinnamaldehyde (0.05%) (antimicrobials)	Cucumber	Antifungal activity (*Fusarium solani*)	[85]
Chitosan (1%) (Coating)	Nano-silica (0.05%) (anti-browning agents, antioxidants, antimicrobials)		Decrease in PPO activity and browning; Reduced weight loss and TA	[86]
Chitosan and alginate (Coating)	Pomegranate peel extract (PPE) (anti-browning agents, antioxidants, antimicrobials)	Capsicum	Decrease loss in weight, firmness, color, and ascorbic acid content	[87]
Sodium alginate (Dip coating)	Eugenol (Eug) and Citral (Cit) (anti-browning agents, antioxidants, antimicrobials)	*Arbutus unedo* fruit (red berry)	Decrease microbial growth and weight loss; Improve physicochemical and biochemical parameters: color, firmness, AOC, and sensorial attributes	[77]
Sodium alginate (Dip coating)	Essential Oil extracted from sweet orange (antimicrobials)	Tomatoes	Decrease weight loss up to 3-fold lower than uncoated samples; Decrease bacterial growth; Increase the firmness with up to 33%	[79]
Sodium alginate (Dip coating)	Citral nano-emulsions (anti-browning agents, antioxidants, antimicrobials)	Pineapples	Better color retention, low respiration rate, reduce microbial growth	[81]
Sodium alginate (Dip coating)	CaCl_2_ (antioxidants, antimicrobials)	Rose apple	Significantly reduce the respiration rate and weight loss; Improve total phenolic content and antioxidant activity	[83]
Sodium alginate, konjae glucomannan and starch (Dip coating)	lotus leaf extract (antioxidants, antimicrobials)	Goji berries (*Lycium barbarum* L.)	Reduce decay rate and weight loss; Maintain AA, TA, TSS;	[42]
Modified starch from sweet potatoes (Dip coating)	Cumin essential oil (antimicrobials)	Pears	Suppress the respiration rate and delay the weight loss and maintain flesh firmness	[78]
Starch and nystose (Dip coating)	Nystose (antioxidants, antimicrobials)	Blackberries	Positive effects in delaying the increase in pH, maintaining the firmness and anthocyanin content	[38]
Arabic gum (Dip coating)	Salicylic acid (anti-browning agents, antioxidants)	‘Grand Nain’ bananas	Decrease weight loss; Improve firmness and peel browning index; Maintain antioxidant activity	[63]
Arabic gum, xanthan gum (Coating)	Lemon grass essential oil 1% w/v and carrageenan (antioxidants, antimicrobials)	Strawberries	Decrease weight loss; Increase AA, AOC, and firmness; Maintain TANC and TPC	[66]
*Protein-based matrix (vegetable proteins as: corn zein, wheat protein, soy protein, and animal proteins as keratin, collagen, gelatin, casein, fish myofibril protein, egg white protein, protein whey)*				
Gelatin (5, 6, and 7%) (Dip coating)	Persian gum (3.5, 4, and 4.5%) and 9, 10, and 11% Shellac (antioxidants)	Oranges	Decrease of weight loss; Decrease TA; Increase TPC and AOC; Maintain fruit firmness and glossiness	[75]
Gelatin (Spraying)	Ethanolic Extract of Propolis (PEE) and zein nanocapsules (antimicrobials)	Raspberries (*Rubus idaeus L*.)	Antifungal activity against *P. digitatum* and *B. cinerea* strains; Increase shelf life	[68]
Nano-structured edible coating based on zein (Controlled release coating system)	Resveratrol (anti-browning agents, antioxidants)	Apple slices	Improve color retention; Decrease moisture loss	[58]
Pectin and pullulan (Coating)	*Vitis vinifera* grape seed extract (antioxidants, antimicrobials)	Peanuts	Reduced lipid oxidation and antibacterial activity against *E. coli* and *L. monocytogenes*	[69]
*Mixed formulations or heterogeneous coatings*				
Aloe vera-based gel (Dip coating)		Papaya fruits	Decrease microbial growth rate; Increase TSS, TA, AA, TCAC, TPC, and TFC; Extend the shelf life by 25%.	[41]
Starch and starch-whey protein coatings (Coating)		Plums	Increase the total resistance in the water vapor pathway	[65]

PO-peroxidase; PPO-polyphenol oxidase; PME-pectin methylesterase; TA-titratable acidity; TPC-total phenolic content; TFC-total flavonoids content, TANC-total anthocyanin content; TCAC-total carotenoids content; AOC-antioxidant capacity/content; TSS-total soluble solid; AA-ascorbic acid.
